# Some General Considerations in the Post-Operative Care of Uncomplicated Abdominal Sections

**Published:** 1916-01

**Authors:** Regina Flood Keyes

**Affiliations:** Woman Gynecologist Buffalo General Hospital; Obstetrician Erie Co. Hospital; Obstetrician St. Mary’s Hospital; Clinical Instructor Obstetrics University of Buffalo


### Some General Considerations in the Post-Operative Care of Uncomplicated Abdominal Sections.

By REGINA FLOOD KEYES, M. D.,

Woman Gynecologist Buffalo General Hospital; Obstetrician Erie Co. Hospital; Obstetrician St. Mary's Hospital; Clinical Instructor Obstetrics University of Buffalo.

The general principles regarding the after-treatment of abdominal cases are well known. Upon this post-operative care hinges, not only the patient's best chance of recovery, but the best final functional result. Considerations, not always recognized as important, are the patient's mental condition and surrounding influences during convalescence. To these should be given the same careful investigation as to the workings of all the organs.

In the majority of cases, after a properly conducted aseptic operation, what the patient requires most is to be let alone and allowed to get well. Complete quiet is to be maintained for twenty-four hours and in my opinion morphine should be administered in ¼-grain doses frequently, then replaced by a milder sedative. The posture in bed is of importance and should not be confined to one position and on the morning of second day head rest is to be used. This favors peristalsis and assists materially in relieving flatus and nausea. Changes in posture are safe-guards against hypostatic pneumonia and the mental effect of sitting up increases confidence and acts most favorably.

The degree of nausea and vomiting following the administering of an anesthetic governs the treatment and it may be said, the less ether, the less nausea. In mild cases, no treatment is necessary beyond rest for the stomach. When vomiting persists, it may be treated by the use of milk of magnesia and after twenty-four hours a 0.02 Cocaine, one minim of Carbolic Acid, one or more minims of dilute Hydrocyanic Acid in a small amount of water. This prescription was introduced by the late Dr. Roswell Park and is in use in the Buffalo General Hospital.

Enemas given morning and night will relieve flatus and are preferable to cathartics. The less medication after laperotomies, unless for some special indication, the quicker will the patient's gastro-intestinal canal return to normal.

Cracked ice and water may be given as soon as desired and when the anesthetic nausea has ceased, liquid food may be given. Milk or light broths are best, then follow with farinaceous foods. The appetite may not return for days and it is not necessary to force the feeding. Rectal feeding may be used, but is not generally indicated.

Close attention should be given to the excretions and every measure taken to promote voluntary evacuation of the bladder. If, however, retention does occur, the catheter may be resorted to. More than ordinary care is necessary to prevent infection in this procedure. The passage of flatus and early movement of the bowels is most gratifying to the surgeon and not more than twenty-four hours should elapse before evacuation occurs.

A wound aseptically made usually heals without complications. The dressings should be carefully watched, although too early and too frequent inspection is not advised. Re-dressing is usually done from fifth to seventh days, at which time the stitches are removed and dry sterile gauze applied. During the dressings, the wound or parts surrounding should never be touched by the hands. All manipulations should be done with sterile instruments and sponges and as much care exercised in the preparation of the hands as is employed at the original operation. The use of rubber gloves precludes the danger of infection only as they are properly donned and the general rules of strict aseptis observed.

The stay in bed should be as short as is compatible with wound rest. A properly fitted bandage relieves wound tension, gives a sense of comfort and tends to prevent the occurrence of future hernia. Normal quiet is maintained in this way and the intra-abdominal pressure acts with the extra-abdominal support to affect the rest of the wound and surrounding parts.

Sunlight and fresh air are as essential to recovery of the surgical case as of the medical. The removal of patient, for a few hours every day to the open air, beginning not later than the end of first week, aborts many of the nervous symptoms and hastens convalescence. Reiss of Chicago, Fowler of Brooklyn and Mayo Brothers of Rochester, have their patients out of bed from the third to fifth days after laperotomies. My practice has been, during my entire service of seventeen years at the Buffalo General Hospital, to have my patients out of bed on the fourth to sixth day. From the first the patient should be encouraged to help herself, to be allowed to get out of bed without aid and after the first day out of bed, should walk unassisted. By the tenth day patient should be in condition to leave hospital if residing locally.

The best functional results are dependent largely upon the surgeon's ability to surround patients with healthy mental influences. If allowed to dwell upon the seriousness of their own condition or permitted to discuss the morbid details of similar cases, nervous symptoms usually appear and may result in pronounced hysteria. It is my practice to pledge my patients to abstain from all thought or discussion of their operations and to refuse to listen to details of the surgical experiences of their friends. In the cases where I have had intelligent co-operation in this particular phase of post-operative care, I have had the best functional results.

---

**Pneumonia Inoculation.** G. D. Maynard, Med. Jour. of S. Africa, Sept. In order to determine whether systematic inoculation should be practiced in native mine workers, the following statistics were collected. A total of 55,900 natives were equally divided, half being inoculated. In 10 months, 576 cases of pneumonia developed in the uninoculated, 459 in the inoculated: 20.6 and 16.4 : 1000, respectively. Of the former 78 died, of the latter 61: 13.5 and 13.3 : 1000, respectively. By periods of 4 weeks, after inoculation, the inoculated showed a lessened incidence, as follows: 1st period, 97 against 119 cases in uninoculated; 2d period, 90 against 120; 3d period, 60 against 83; 4th period, 56 against 86; 5th period, 43 against 57. Thereafter, the incidence was slightly greater among the inoculated. With reference to other respiratory diseases and to disease incidence in general, no appreciable difference was noted, although another series suggested that there was a slight diminution of respiratory diseases generally, because many of them were of pneumococcic origin, though not anatomically pneumonia. The question whether repeated inoculation would have a beneficial action is waived.

---

**Specialism.** P. R. W., a student of the Harvard Medical School, Boston M. and S. Jour., Nov. 18, has canvassed the graduates of three recent classes and compiled statistics of 86 heard from. 27 of these had secured essentially medical hospital interneships, 26 surgical, 27 mixed services, 1 a pathologic service and 5 none. 69 gave replies as to nature of private practice which may be interpreted as general practice, 8 surgery, 2 surgery and obstetrics, 1 eye, ear, nose and throat, 5 laboratory work, 1 not practicing. He calls attention by statistics which we do not cite, as they are susceptible of various constructions, to the fact that the interneship does not necessarily fit the individual for the work in which he ultimately engages.

---

**Resection of Tuberculous Lymph-nodes.** In the October Clinics, Dr. John B. Murphy incidentally mentions that it is now eight years since they performed the last dissection of the neck for tuberculous glands. They use tuberculins and do no surgical operation except occasionally to drain if a mixed infection is present.

